# The role of infections in the pathogenesis and course of multiple sclerosis

**DOI:** 10.4103/0972-2327.64622

**Published:** 2010

**Authors:** Siddharama Pawate, Subramaniam Sriram

**Affiliations:** Department of Neurology, Vanderbilt University Medical Center, Nashville, TN, USA

**Keywords:** Oligodendrocyte, cellular immunity, toll like receptors, oligoclonal bands, molecular mimicry

## Abstract

Interplay between susceptibility genes and environmental factors is considered important player in the genesis of multiple sclerosis (MS). Among environmental factors, a role for an infectious pathogen has long been considered central to the disease process. This opinion has support both from epidemiological data and the findings of immunological abnormalities in spinal fluid that reflect an immune response to an as yet undetermined antigen, possibly a pathogen, in the cerebrospinal fluid. Our review will outline the current understanding of the role of infection in the causation and progression of MS. We will review the data that point to an infectious cause of MS and consider the specific agents Chlamydophila (Chlamydia) pneumoniae, Human Herpes Virus 6, and Epstein-Barr Virus, that are implicated in either the development or progression of MS.

## Introduction

The cause of multiple sclerosis (MS) is not known and the role of environment and the immune response is well accepted as key players in the disease process. Current opinion favors the notion that MS is an autoimmune disease directed against self-neural antigens.[1] The autoantigens that may be responsible for the autoimmunity have remained elusive.[2] Much of the speculation that has driven the notion of autoimmunity in MS is due the similarities between MS and the mouse model of experimental autoimmune encephalitis.[2] There have been a number of clinical and epidemiologic observations that point to an infectious process involved in MS. Our review will focus on the role of potential pathogens that have been implicated directly in the initiation and development of MS and the difficulties in ascertaining a causal connection between pathogen and disease.

## Epidemiology

MS affects approximately 1,000 000 people between 17 and 65 years old worldwide.[3] Because MS begins in young adulthood and is responsible of physical deficits that are long lasting, the socioeconomic impact of MS is probably greater than those of Alzheimer's disease and stroke.[4] While the disease has its highest prevalence in Europe and North America (temperate regions), there is indirect evidence that the incidence of the disease is increasing in tropical regions and in the developing world.[5] While the prevalence of MS in North America ranges between 30/100,000 and 125/100,000, the prevalence in tropical regions including the Indian subcontinent is approximately 1.33/100,000,[6] a considerably lower rate when compared to Europe and North America. A study from West Bengal estimated a male: female ratio of 1:1.15 and a mean age of onset 31.83 years in men and 29.11 years in women.[7] MS was reported to make up 0.85% of admissions to neurology departments of major medical centers in western India, comparable to other parts of India[8] suggesting that MS is still a rare disease in the Indian subcontinent.

## Clinical features

The two core clinical features of MS are relapses, presumed to result from acute inflammatory demyelination, and progression of neurological disability, as a result of inflammatory demyelination, gliosis, and axonal loss.[9] The clinical presentations of MS are divided into four broad subtypes. In 80% of patients, MS presents as relapsing remitting disease (RRMS) that usually lasts for 15 years, characterized by exacerbations and remissions, and partial response to immunomodulatory therapy.[10] The disease then takes on a more insidious course with a monotonous progressive loss of motor functions and is referred to as secondary progressive disease (SPMS).[11] In 15% of patients, MS is progressive from onset (primary progressive MS);[12] nonetheless, this disease subtype is similar to secondary progressive MS.[13] In a smaller subset of patients who begin with primary progressive symptoms, show clinical relapses and are called progressive relapsing MS.[14]

## Pathology

Irrespective of the clinical presentation, the one constant feature of all the different clinical subtypes involves loss of myelin (demyelination) and damage to the underlying axons. The central histological feature of MS is the MS plaque,[15] which is described as acute, (prominent inflammatory cells and activated microglial cells), subacute, and chronic (paucity of immune cells and prominent gliosis). The lesions are typically located in the optic nerves, periventricular areas, cerebellum and floor of the fourth ventricle, and in cervical spinal cord. Lucchinetti and her coworkers have suggested that the MS lesions are heterogeneous.[16] They have categorized four lesion types in MS. Type 1 and 2 lesions are inflammatory in nature and resemble that seen in autoimmune models of CNS demyelination, while Type 3 and 4 lesions show degeneration and apoptosis of oligodendrocytes in the absence of inflammatory cellular infiltrate (primary oligodendrogliopathy). Although harder to detect using MRI, demyelinating lesions are seen within the grey matter as well.[17,18] Within the demyelinating plaque, there is loss of both myelin, oligodendrocytes, and eventually axons. The destruction of axons, although not as prominent as loss of myelin, is nonetheless an important component of the pathology and disability of MS.[19,20]

## Evidence implicating infection in the etiology of MS

Epidemiological data and the inflammatory nature of the lesions have driven the research enterprise in search of a pathogen in MS. Most of the investigations were pursued to substantiate a viral cause for MS although this has not precluded other non-viral pathogens. To satisfy a causal association between MS and an infectious agent, the pathogen should ideally (a) cause a chronic inflammatory disorder of the CNS, (b) preferentially reside within the CNS and undergo periods of activation and quiescence, and (c) should cause demyelination.

## Infections and chronic neurological disease

Herpes Simplex 1 and 2 are well-known pathogens that reside within the neurons in the CNS.[21] They can cause a relapsing remitting disease characterized by the formation of herpetic vesicles in the distribution of the trigeminal nerve, but do not cause demyelination. Many chronic bacterial infections can reside within the CNS and lead to periodic activation and cause widespread pathology. The spirochetes, *Treponema pallidum* and *Borrelia burgdorferi*, cause neuro-syphilis and Lyme disease, respectively.[22] *T. whipplerii*, which causes Whipple's disease, can cause chronic neurological manifestations in some patients.[23] However, none of these bacterial pathogens are known to cause CNS demyelination. JC virus, a papovavirus, can infect oligodendrocytes and cause demyelination.[24] In the majority of cases, progressive multifocal leukoencephalopathy (PML), the clinical syndrome that JC virus infection causes, there is widespread demyelination, but minimal inflammatory response, and no development of features typical of inflammatory plaque. HTLV-1[25] causes a chronic demyelinating disease of the CNS, tropical spastic paraparesis, characterized by progressive paraparesis and extensive inflammatory demyelination, and fits most of the requisites of inflammatory demyelination seen in MS. HTLV-1 is caused by a retrovirus, and efforts to identify a retrovirus in MS have not been forthcoming. Most of the focus has been on identifying a viral agent, in part because, only viruses have been known to infect CNS and cause demyelination.

Animal models of infectious demyelination have been few, and most of the studies have over the years focused on the demyelinating disorder seen following intracerebral injection of Theiler's virus.[26] Although other viruses, including Borona and JCM, have been studied, these models do not cause chronic demyelinating disease. Theiler's virus, a single-stranded RNA virus and a member of the Picornaviridae family, causes a chronic progressive neurological disease in susceptible mice strains. Inoculation of TMEV directly into the brain initially causes an acute disease resembling polioencephalomyelitis, and this is later followed in susceptible strains by a widespread demyelinating disease in the spinal cord attendant with paralysis of limbs. While the TMEV model offers an immunopathogenic mechanism by which an infectious agent can cause demyelination, the necessity of intracerebral inoculation to induce disease makes the relevance of the disease less pertinent to the clinical syndrome.

## Epidemiological observations

Epidemiological evidence for infection in MS comes in two forms, geographical distribution and isolated epidemics of MS. The prevalence of MS increases with distance away from the equator.[27] In North America, the northern regions were settled mainly by people of Scandinavian origin who may be highly susceptible to MS, and this was thought to be part of the explanation.[28] However, a similar gradient was also found in Australia, where the population distribution is relatively homogeneous.[29] The variability may therefore be attributable to environmental factors. Migration studies showed that migration from low MS areas to high MS areas before the age of 15 increased the risk of MS and the reverse was true as well.[30,31] Ethnicity may confer some degree of protection against the increased risk. Thus, in North America and the UK, MS increases among immigrants from India, while it remains low in immigrants from East Asia.[32]

Among the "epidemics" of MS, the Faroe island epidemic is probably the best studied,[33] with others being reported in Iceland[34] and Shetland-Orkneys.[35] Faroe Islands, off the coast of Denmark, had no reported cases of MS until 1940 when British troops were stationed on the islands. Between 1943 and 1990, 54 cases of MS were reported, occurring in 4 waves of 21, 10, 10, and 13 cases. It is speculated that the British troops introduced a new infectious agent into the isolated island, leading to the development of MS. However, there are considerable questions as to the validity of the epidemics,[36] because it is difficult to ascertain the true onset of the disease and the small number of cases makes it difficult to distinguish from a chance occurrence. The lack of similar 'epidemics' in other places where previously isolated populations were exposed to British troops also argued against an MS epidemic.[37]

Two models have been proposed to explain the epidemiological data. According to the 'prevalence' model, MS occurs with the most frequency where the infectious agent is most widespread.[38] In contrast, the 'polio' hypothesis posits that a widespread infection during childhood has a protective effect, whereas a primary infection after puberty, which is more likely to happen in areas with low abundance of the infectious agent, is more likely to cause MS.[39] The "hygiene hypothesis"[40] is a more generalized version of the 'polio' hypothesis and is supported by data that indicate that children in areas where MS is rare are more likely to show evidence of childhood viral infections, the higher prevalence of MS where sanitation is good, and the increased occurrence of MS in people of higher socioeconomic status.

While the epidemiological studies have not delineated a specific infectious agent, they do indicate the presence of an age-related susceptibility to acquiring an infection that triggers MS in genetically prone subjects.

## Biochemical evidence for infectious etiology of MS

The most persuasive argument for the role of an infectious agent is in the profile of the immunoglobulins in the CSF. Following isoelectric focusing of immunoglobulins obtained from CSF and serum, immunoglobulins in the CSF but not serum focus to the cathodal regions of the gel in bands. These Ig bands (also referred to as oligoclonal bands) are present mainly in the CSF and absent in the serum. Oligoclonal bands are seen in 95% of patients with MS.[41] In addition, there is increased synthesis of Ig (elevated IgG index) suggesting an amplified production of Ig within the CNS compartment.

Oligoclonal bands are a common feature of almost all infectious disease of the CNS. The antibody response has been shown to be directed against infectious antigens in HTLV myelopathy,[42] neurosyphilis,[43] and Lyme disease.[44] Rarely, oligoclonal bands are also seen in non-infectious disorders such as neurosarcoidosis,[45] but in most non-infectious disorders, an underlying immune disorder has been recognized. There has been considerable interest in the identifying the putative antigenic targets of the immunoglobulins present in the CSF of MS patients. Thus, far evidence for a particular pathogen or a family of pathogens is lacking.

We have presented evidence that at least in a subset of MS patients, adsorption of chlamydial antigens by the immunoglobulins present in the CSF suggests the presence of antibodies to *C. pneumoniae* in patients with MS.[46]

## LPS-induced model of CNS demyelination-relevance to infection

Direct injection of the LPS into spinal cord results in the development of a chronic demyelinating lesion. These lesions show an early infiltration of lymphoctyes that disappear followed by the activation and infiltration of macrophages.[47] After a delay of 5-7 days and extending up to 21 days, following disappearance of T cells, there was an increase in the size of the area of myelin loss and a well-defined area of demyelination. In addition, detailed pathological studies of the early demyelinating lesions in areas that received LPS show loss of myelin-associated glycoprotein (MAG), in the paranodal areas very reminiscent of Type III MS lesions.[16] These lesions induced by the direct injection of LPS are very similar to the modeled proposed by Barnett and Prineas[48] and type III lesions as classified by Lucchinetti.[16] The earliest changes shared by all newly forming lesions include early loss of myelin-associated glycoprotein (MAG), oligodendrocyte apoptosis, microglial activation, and virtual absence of infiltrating lymphocytes. The ability of LPS to induce pathological changes similar to those seen in MS is intriguing for an infectious hypothesis.[49]

## Mechanisms of infection-induced demyelination

Several mechanisms have been proposed by which infections can cause demyelination and include both direct and indirect mechanisms. Thus, a virus can infect oligodendrocytes leading to its lysis or apoptosis, with consequent demyelination. This is seen in PML, where infection by the JC virus leads to caspase activation in oligodendrocytes, leading to their apoptosis, and in the TMEV model, where productive viral infection leads to lysis of oligodendrocytes, by activation of cytotoxic T cells.[24,26] Viral infection can also lead to induction of an autoimmune response by molecular mimicry or bystander activation.[50] In the molecular mimicry model, shared antigenic determinants between putative infectious pathogens and myelin antigens in a genetically susceptible individual lead to the development of autoreactivity and ultimately autoimmune demyelination. In the bystander activation model, microbial infections lead to significant activation of antigen-presenting cells (APCs) such as dendritic cells. These activated APCs could potentially activate preprimed autoreactive T cells, which can then initiate autoimmune disease (bystander activation of autoreactive immune T cells).

## Proposed infectious agents in MS [Table 1]

**Table 1:** Partial list of infectious agents implicated in MS, and the evidence for implicating them. (See the text for details)

Pathogen	Association with MS	Reference
Chlamydia pneumoniae	Isolation of Cpn from CSF; improvement with antibiotic treatment	53
	PCR identified Cpn outer membrane protein gene; ELISA showed 86% of MS patients had Cpn antibodies in their CSF	46
	Failure to identify Cpn by PCR or culture in CSF and autopsy specimens of MS patients	54,55
Human herpes virus - 6	Identification of a DNA fragment homologous to major DNA binding protein gene of HHV-6 in CSF of MS patients	61
	Increased IgM antibody titers to HHV-6 in MS patients compared to controls	62
	Presence of HHV-6 DNA in 57.8% of MS plaques and 15.9% of normal brain samples	63
	Failure to detect HHV-6 DNA in CSF from MS patients	64
	No differences in antibody responses to HHV-6 in MS and other neurological diseases	65
Epstein-Barr virus	Seropositivity in up to 95% of the general population but almost 100% of MS patients	69
	Presence of CD-8 responses to EBV but not CMV, in early MS	72
	Demonstration of EBV in infiltrating B-cells in 21/22 patients with MS but not other inflammatory neurological diseases	74
Retrovirus	Detection of LM7 retroviral RNA in CSF	77

### Chlamydophila pneumoniae

Chlamydiae are gram-negative, obligate intracellular pathogens known to cause chronic infections. Cpn appears to be ubiquitous and has been implicated in several chronic diseases including atherosclerosis, vasculitis, and Alzheimer's disease.[51,52] Cpn were implicated in the pathogenesis of MS after Cpn were isolated at Vanderbilt from the CSF of a patient with rapidly worsening MS, who improved after being treated with antibiotics.[53] This was followed by a study using tissue culture, isolated Cpn from 64% of CSF samples from MS patients and only 11% of OND controls. PCR identified Cpn outer membrane protein gene in the CSF of 97% of MS patients and 18% of controls, and ELISA showed that 86% of MS patients had Cpn antibodies in their CSF.[46] The results were called into question after other centers failed to identify Cpn by PCR or culture in CSF and autopsy specimens of MS patients,[54,55] but this could be attributed to technical variations.[56] Since then, several centers have continued to report the association of Cpn with MS while others have not found one. In 2006, Bagos *et al*.[57] performed a meta-analysis of 26 studies involving 1332 MS patients and 1464 controls, and concluded that "Even though the presence of Cpn is clearly more likely in MS patients, these findings are insufficient to establish an etiologic relation."

### Human Herpes Virus-6 (HHV-6)

Along with Epstein-Barr virus (discussed below), HHV-6 is one of the two herpes viruses associated with MS. HHV-6 is ubiquitous, with a seropositivity of more than 90% in the general population. Infection usually occurs early in childhood, causing exanthema subitum.[58] This early infection is consistent with the notion that exposure to an infective agent causing MS occurs early in life.[59] HHV-6 is also neurotrophic and primary infection occasionally results in meningitis, encephalitis, and febrile seizures.[60] It is also thought to achieve latency in the CNS and reactivate during periods of stress. Association between HHV-6 and MS was proposed after representational difference analysis showed the presence of a DNA fragment homologous to major DNA binding protein gene of HHV-6.[61] Since then, some groups have found increased IgM antibody titers to HHV-6 in MS patients compared to controls, indicating recent infection.[62] Cermelli *et al*.[63] found the presence of HHV-6 DNA in 57.8% of MS plaques and 15.9% of normal brain samples, a result highly statistically significant (*P*< 0.0005). However, other groups failed to detect HHV-6 DNA in CSF from MS patients,[64] and found no differences in antibody responses to HHV-6 in MS and other neurological diseases.[65] One study found HHV-6 DNA in cell-free CSF in 11% of MS patients, 7% of HIV and 0% in other neurological diseases. When cells in the CSF were included, the numbers rose to 39%, 41% and 29%, suggesting that the viral DNA was present in the cells that had infiltrated the CSF as a response to inflammation.[66] Thus, the role for HHV-6 in MS remains inconclusive.

### Epstein-Barr virus

EBV is a lymphotropic virus that causes asymptomatic infections in children. In adolescents and adults, EBV infection is likely to cause infectious mononucleosis in almost half the cases.[67] EBV has been associated with several autoimmune diseases, including systemic lupus erythematosus, Sjogren's syndrome, Hashimoto's thyroiditis, and rheumatoid arthritis.[68] EBV seropositivity is found in up to 95% of the general population but almost 100% of MS patients,[69] raising possibilities that EBV may be associated with MS. The ubiquity of EBV makes it difficult to ascertain this association, but Ascherio and Munger[70] analyzed the data from 13 studies and found that compared to people infected with EBV in childhood, those infected in adolescence and adulthood had a 2-3 fold higher likelihood of getting MS, whereas those who were EBV negative were 10 times less likely to have MS. This suggested a role for EBV in accordance with the hygiene hypothesis. However, attempts to find EBV in the brains of MS patients have been unsuccessful in the past.[71] More recently, Jilek *et al*.[72] showed the presence of CD-8 responses to EBV but not CMV, in early MS, while Zivadinov *et al*.[73] correlated grey matter atrophy in MS with anti-EBV antibody responses. These studies and the demonstration of EBV in infiltrating B-cells in 21/22 patients with MS but not other inflammatory neurological diseases[74] strengthen the case for EBV as a factor in MS. Supportive validation of these studies from other laboratories is awaited.

### Other agents

Human endogenous retroviruses (HERV) have been implicated in MS.[75,76] Another retrovirus, LM7, has been found in some patients with MS.[77] Coronaviruses have also been associated.[78] The precise roles of any of these agents in MS remain to be elucidated.

## Infections and MS relapses

In addition to their possible role as initiators of the disease process, infections have been thought to trigger relapses in RRMS. In a pioneering study, Sibley *et al*.[79] studied viral infections and their association with MS relapses. They followed up 170 patients at monthly intervals for an average of 5.2 years. They found that during periods of viral infections (2 weeks before to 5 weeks after), the annualized rate of relapses was 3-fold higher. Approximately 9% of infections were related temporally to exacerbations, while 27% of exacerbations were infection related. Andersen *et al*.[80] noted that relapses were less frequent in the summer, when viral respiratory and gastrointestinal infections were less common. Rapp *et al*.[81] studied bacterial infections and their relation to relapses and found that 50% of patients with relapses had bacterial infections, compared to 11% of MS patients without relapses. Buljevac *et al*.[82] incorporated brain MRI into their study of infections and relapses. They followed up 73 patients with RRMS for an average of 1.7 years. They found that during the at-risk period (2 weeks prior to 5 weeks after an infection), the annualized rate of relapses increased 2.1-fold. They further found an increase to 3.8-fold for sustained relapses (lasting more than 3 months) for relapses during the at-risk period. They found no differences in the gadolinium-enhancing lesions on brain MRI between relapses during and independent of infections. On the other hand, Correale *et al*.[83] studied the effects of systemic infections (viral and bacterial) on MS relapses, brain MRI activity, and cytokine production in 60 patients with RRMS. They found an approximately a 3-fold increased risk of relapses during infections (2 weeks prior to 5 weeks after an infection), with a peak at 2 weeks after infection. They performed three sequential MRIs, at weeks 0, 2, and 12 following the onset of a relapse. They found an increase in the number of gadolinium-enhancing lesions on brain MRI at 2 weeks, during the infection-associated relapses compared to relapses outside the at-risk period. They also found increased myelin-specific T-cell activation during infection-associated relapses. There were no differences between viral and bacterial infections. The mechanisms by which infections can trigger relapses are also thought to involve bystander activation or molecular mimicry.

## Conclusions

A review of current literature shows that infectious agents remain a viable trigger for the initiation of the disease pathogenesis in MS. However, the quest for the identification of a specific bacteria or virus has not yet succeeded. Viral and bacterial infections have also been shown to be associated with disease exacerbations in RRMS.

There are a number of barriers to make a conclusive argument for a causal connection between MS and an infectious agent. First, within the community of MS scientists there is disagreement as to whether MS represents a syndrome or in fact one disease. Controversy persists as to the heterogeneity of MS lesions between individuals with the disease. If so, it might indicate that one disease one pathogen hypothesis may be difficult to reconcile and the clinical syndrome of MS will be partitioned into different entities. This has already happened with the identification of antibodies to aquaporin IV, making NMO an entirely different disease and not a variant of MS. Similarly, Susac's syndrome is considered a microangiopathy with clinical and radiological similarities to MS. On the other hand, infectious disease can have varied pathological presentations, depending upon the acuity of the disease. In syphilis, the acute lesions are characterized by the marked lymphocytic infiltration, while the chronic CNS disease shows the formation of gummata and neurodegeneration with a paucity of inflammation.

The second hurdle is the problem in providing a causal connection between pathogen and disease, especially when the pathogen can be ubiquitous. In these situations, the commonly held postulates to show a connection between pathogen and disease become obsolete and only a clinical therapeutic trial, based on "clinical hunch" can be definitive. The story of a connection between *H. pylori* and gastric ulcers is a case in point.[84] In other instances such as the association between viral infection and tumors, the association between Hepatitis B and liver carcinoma was only deduced in the reduction of hepatic cancer following vaccination against Hepatitis B. The possibility of a single unitary agent responsible for MS is still a distinct possibility. As noted by Lipton *et al*.,[85] molecular approaches that do not depend upon prior knowledge of the nature of an infectious agent, such as recombinant antibodies, phage display and cDNA expression libraries may facilitate the discovery of an infectious agent. It is more likely that the disorder we recognize as MS may turn out to be more a syndrome than a single disease. If this were so, a subset of patients an infectious agent may be the agent that both initiates and sustains the disease.
